# Selenium Nanoparticles Induce Potent Protective Immune Responses against *Vibrio cholerae* WC Vaccine in a Mouse Model

**DOI:** 10.1155/2020/8874288

**Published:** 2020-12-30

**Authors:** Zahra Raahati, Bita Bakhshi, Shahin Najar-peerayeh

**Affiliations:** Department of Bacteriology, Faculty of Medical Sciences, Tarbiat Modares University, Tehran, Iran

## Abstract

The aim of this study was to evaluate the efficacy of selenium nanoparticle (an immune booster) and naloxone (an opioid receptor antagonist) as a new adjuvant in increasing immune responses against killed whole-cell *Vibrio cholerae* in a mouse cholera model. The Se NPs were synthesized and characterized by UV-visible, DLS, and zeta potential analysis. The SEM image confirmed the uniformity of spherical morphology of nanoparticle shape with 34 nm in size. The concentration of the Se NPs was calculated as 0.654 *μ*g/ml in the ICP method. The cytotoxic activity of Se NPs on Caco-2 cells was assessed by the MTT assay and revealed 82.05% viability of cells after 24 h exposure with 100 *μ*g/ml of Se NPs. Female BALB/C mice were orally immunized three times on days 0, 14, and 28, and challenge experiments were performed on immunized neonates with toxigenic *V. cholerae*. Administration of Se NP diet led to significant increase in *V. cholerae*-specific IgG and IgA responses in serum and saliva and caused protective immunity and 83.3% survival in challenge experiment against 1 LD50 *V. cholerae* in a group receiving diet of Se NPs compared with other groups including Dukoral vaccine. The IL-4 and IL-5 were significantly increased in response to WC+daily diet of Se NPs with or without naloxone. Naloxone proved no effect on IL-4 and IL-5 increase and is proposed as null in the cytokine and antibody production process. These results reveal that daily diet of Se NPs could efficiently induce immune cell effectors in both humoral and mucosal levels.

## 1. Introduction

Cholera disease is an acute diarrhea with an old history caused by *Vibrio cholerae*, a gram-negative and motile bacterium. Cholera toxin (CT) is responsible for the watery diarrhea which is produced by toxigenic *Vibrio cholerae* [1]. Since the year 1817, there have been 7 pandemics of cholera in the world. Cholera pandemic was caused by *V. cholerae* biotypes O1 and 0139 [1]. Today, cholera remains a global problem in many parts of the world, and from 2000 to 2016, 3.4 million cholera cases and 65600 deaths occurred worldwide [2].

Vaccination is a complementary strategy for prevention and control of cholera diseases alongside providing sanitation and safe water. Mucosal surfaces are considered the first line of host-microbe interactions that cause the establishment and maturation of mucosa-associated lymphoid tissues and affect the induction and initiation of innate together with acquired mucosal immune responses. Mucosal vaccination has some functional and practical advantages, including inferior costs and no pain and risk of needle-stick injuries and subsequent blood-borne diseases. Moreover, mucosal vaccination can also induce both humoral- and cell-mediated immune responses in both systemic and mucosal compartments and can efficiently induce long-lasting B cell- and T cell-associated memory [3, 4]. Accordingly, oral delivery can induce production of antigen-specific sIgA in the gastrointestinal tract, salivary glands, and mammary glands [3]. Several antigens of *V. cholerae* have been studied as inducers of the immune system against bacteria, but these antigens create only a short-term protection if applied alone [5]. Currently, two types of oral killed cholera vaccine are available and have global license: killed whole-cell (WC) *Vibrio cholerae* O1 plus recombinant CTB (Dukoral) and killed whole-cell bivalent O1 and O139 (Shancol). Oral cholera vaccines have been instrumental in controlling disease in endemic areas, but the protective immunity initiator by these vaccines is low-level and short-term [6]. However, in Russia, an oral vaccine against cholera diseases, other than the global commercial cholera vaccines, is being used which is a combination of cholera toxoid and O-antigens that has been shown to induce a high protective immune response and is stable in human for 6 months [7].

With the development of nanoscience, recent studies on vaccines have led to the use of nanoparticle in vaccine formulations or as adjuvants alongside vaccine components. Selenium element is one of the rare and essential minerals in the body with various functions such as anticancer and antiviral and is part of the antioxidant enzymes [8–11]. Selenium in the nanoscale has attracted considerable attentions in the present era due to the high bioavailability and low toxicity. Selenium nanoparticles (Se NPs) in the laboratory are made chemically or biogenically and have been proven functional in stimulating the immune system through various pathways, such as innate immunity, T cells, and NK cells [12].

Furthermore, opioids are in fact a collection of opiate-like substances. Naloxone (Nal) is an opioid antagonist, previously studied as an effector to shift immune response to cellular immunity by Th1 response [13, 14].

The aim of this study was to examine the adjuvant activity of naloxone and Se NP and increase immune responses and protective immunity against *Vibrio cholerae* whole cells in challenge experiment of cholera in a mouse model.

## 2. Materials and Methods

### 2.1. Ethical Consideration

The study was evaluated and approved by the research ethics committee of Tarbiat Modares University under IR.MODARES.REC.1397.068 approval ID before it began. All methods were carried out in accordance with relevant guidelines and regulations in manuscript which have been reviewed by the animal care committee of Tarbiat Modares University. Personnel involved in this research were trained and successfully passed the animal care courses.

### 2.2. Bacterial Strains

*V. cholerae* ATCC 14035, a classical O1 serotype, was used for oral immunization and challenge experiments. Approximately 10^8^ CFU (colony-forming unit) of killed whole-cell *V. cholerae* was used for mouse immunization. *V. cholerae* ATCC 14035 in a 50% lethal dose was used in neonatal mouse challenge.

### 2.3. Inactivation of *V. cholerae*

*V. cholerae* cells were cultured overnight in BHI agar at 37°C, and a fresh 18-hour subculture was prepared in LB broth. Bacterial cells were washed three times with PBS and diluted to 10^9^ cells/ml and killed with 1% formaldehyde-PBS for 2 h. Cells were washed twice with PBS and stored at 4°C for future use [15].

### 2.4. Synthesis of Selenium Nanoparticles (Se NPs)

Se NPs were synthesized by the reduction of sodium selenite by the ascorbic acid. Ascorbic acid (Merck, Germany) in a concentration of 58.13 mM was slowly added to 1.2 mM Na_2_SeO_3_(5H_2_O) (Merck, Germany) under a stirrer at 1300 rpm. The solution was washed twice and resuspended in 1 ml deionized water. Tween 20 (30 *μ*l/20 ml) was used to prevent the aggregation of selenium nanoparticles during the synthesis procedure [16].

### 2.5. Characterization of Se Nanoparticles

The identity of Se NPs was confirmed by UV-vis absorption spectroscopy (PerkinElmer, Lambda 25) at 200-500 nm. The size distribution of Se nanoparticles and the surface charge of the nanoparticles (zeta potential) were determined by the dynamic light scattering (DLS) analysis using Malvern Zetasizer Nano-ZS instrument.

The morphology and particular size of Se NPs were determined by scanning electron microscopy (SEM, FEI Company of USA, model Quanta 200). Nanoparticles in MilliQ water were allowed to slowly dry on a glass slide and covered with a layer of gold metal before picture documentation.

Selenium content of the nanoparticles was determined by Inductively Coupled Plasma-Atomic Absorption Spectroscopy (ICP-AAS). Acid digestion of nanoparticles was carried out using a solution of 2% nitric acid. Selenium standards were then prepared from sodium selenite salt at concentrations of 1-100 ppm, and the concentration of nanoparticles was measured based on the standard concentration of selenium.

### 2.6. Cell Culture and Assessment of Cytotoxicity of Se NPs

The toxicity of Se NPs was calculated by the MTT assay. Caco-2 cell lines were purchased from Iranian Biological Resource Center. The Caco-2 cells were cultured in DMEM containing fetal bovine serum (10%) and penicillin and streptomycin at 37°C at 5% CO_2_. Cells were centrifuged for 5 min at 1500 rpm and counted on the Neubauer chamber with 1/2 trypan blue, and the appropriate volume of cell suspension (10^5^ cells) was seeded to a 96-well plate [17]. The cells were incubated with 50-200 *μ*g/ml Se NPs for 24 and 48 h after which 100 *μ*l DMEM medium containing reconstituted MTT (10 mL) was added and the plates were returned to the incubator. After 4 h, the medium was removed and 100 *μ*l of detergent reagent (DMSO) was added to solubilize formazan crystals. Optical densities at 570 nm were read, and the percentage of live cells was calculated relative to the control cells.

### 2.7. Animals

The immune responses were evaluated in 4-6-week-old female BALB/C mice purchased from Pasteur Institute of Iran. The mice were kept in 8 groups in separate cages under normal condition, and each group consisted of 6 mice [18]. The mice were acclimated for 5 days before beginning the experiment, and during this time, the animals were exposed to the standard condition of temperature and humidity. Experiment on animals was carried out in accordance with relevant guidelines and regulations of the Institute of Laboratory Animal Resources [19].

### 2.8. Mouse Immunization

Animals were kept fasted for 5 h and subjected to gastric acid neutralization with 5% sodium bicarbonate. The immunization of mice was performed on days 0, 14, and 28 [20, 21]. Mouse groups are summarized in Table 1.

WC and Se NPs were used by the intraintestinal route with standard gavage. In the group that diet of selenium was used, mice received a daily dose of 100 *μ*g of selenium NPs on days 0 to 42.

Naloxone has no absorption from the gastrointestinal tract, and according to the instructions, the naloxone drug was injected.

### 2.9. Sample Collection

Two weeks after each immunization, on days 0, 14, 28, and 42, blood and saliva samples of mouse groups were collected [20, 21]. Blood samples were incubated at 4°C for 60 min and centrifuged at 10000 rpm for 10 min, and serum was separated. The saliva sample was collected with a sterile swab from the mouth and was stored in 100 *μ*l of sterile PBS. Saliva and separated serum were stored at -20°C for further analysis.

### 2.10. Measurement of Antibody Responses

Enzyme-linked immunosorbent assay (ELISA) was used to measure the total IgG antibodies in serum samples. ELISA plates were coated with 10^8^ CFU of whole-cell *V. cholerae* as an antigen and incubated at 4°C overnight. Nonbinding sites were blocked with 2% BSA at 37°C for 2 h. Plates were washed three times with PBS-0.05% Tween 20 (PBST), and 100 *μ*l of sera in 1/100 through 1/12800 dilutions was added to each well and incubated at 37°C for 2 h. After washing steps, 100 *μ*l of peroxidase-conjugated goat anti-mouse IgG (Sigma-Aldrich) diluted in PBST (1 : 10000) was added to each well. Plates were incubated for additional 1.5 h at 37°C. After washing 3 times, 100 *μ*l of tetramethylbenzidine (TMB) was added to wells and incubated in the darkness for 30 min. The reaction was stopped by 1 M H_2_SO_4_, and the absorbance values were read by using an ELISA reader (Labsystems, model no. 352) at 450 nm [22]. An IgA antibody in saliva and serum samples was evaluated in the same procedure with goat anti-mouse IgA in 1/10000 dilution (SIGMA Aldrich) as the secondary antibody. Serum and saliva dilutions for IgA antibody evaluation was 1/100 through 1/12800 dilutions for serum and 1/25-1/800 dilutions for saliva.

### 2.11. Cytokine Assays

Cytokine production (IL-4, IL-5) was assessed on blood samples by the ELISA method using the R&D Systems kit. Briefly, 100 *μ*l capture antibody was added to each well of plate and was incubated at room temperature overnight. Plates were washed three times with wash buffer (PBS-0.05% Tween 20). Then, Reagent Diluent was added to each well and was incubated for 1 h. Following washing, 100 *μ*l serum was added to wells and was incubated for 2 h. After washing, a detection antibody was added and was incubated for 2 h and then was washed again. Streptavidin-HRP was added and was incubated for 20 min. 100 *μ*l TMB was added and was incubated in a dark place for 20 min. Stopper solution (2N·H_2_SO_4_) was added, and the absorbance was measured with an ELISA plate reader at 450 nm.

### 2.12. Challenge Experiments

To assess protective immunity, challenge experiments were performed on 3- to 5-day-old unimmunized pups. The experiment included 8 groups, and each consisted of 6 pups. The neonates were kept separated from dams at 30°C, and their standard condition of temperature and humidity was controlled every 4 h for 48 h. Death and health of mice were recorded every 4 h. A total of 50 *μ*l, containing 25 *μ*l of immune serum from immunized mice on day 42 plus 25 *μ*l of LB broth containing 10^6^ CFU of *V. cholerae* ATCC 14035, was given to pups orally [22, 23]. The measurement criteria of the challenge were evaluation of animals' resistance in the control group compared with animals injected with immune serum followed by evaluation of elapsed time of death of resistant animals compared with the control group.

### 2.13. Statistical Analyses

The ANOVA test and *t*-tests were used to assess the significant differences. In neonate challenges, we analyzed survival curves by the log-rank test. The significant values were considered less than 0.05. All antibody responses and cytokine measurements were performed in triplicate, and the results were presented as the mean of experiments ± standard deviation.

## 3. Results

### 3.1. Characterization of Se NPs

**Figure 1:**
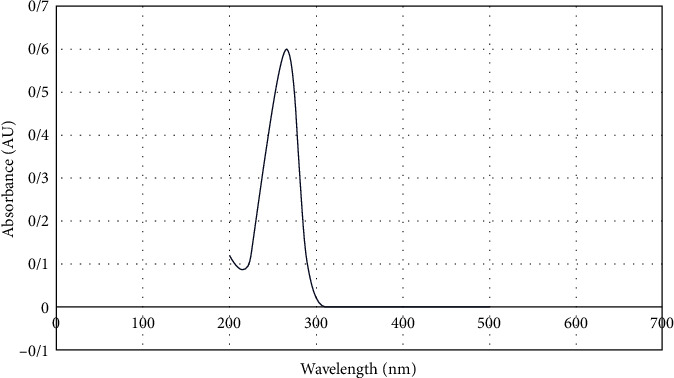
UV-vis spectrum of Se NPs.

The UV-visible absorption spectra of nanoparticle showed a maximum absorption value at around 260 nm (Figure 1). The appearance of a sharp extinction peak around the specified wavelength with low FWHM (full width at half maximum) confirmed the conversion of selenite ions into Se NPs and monodispersity of the nanoparticles. The characteristic orange color of Se NP colloidal suspension was also visually evaluated.

**Figure 2:**
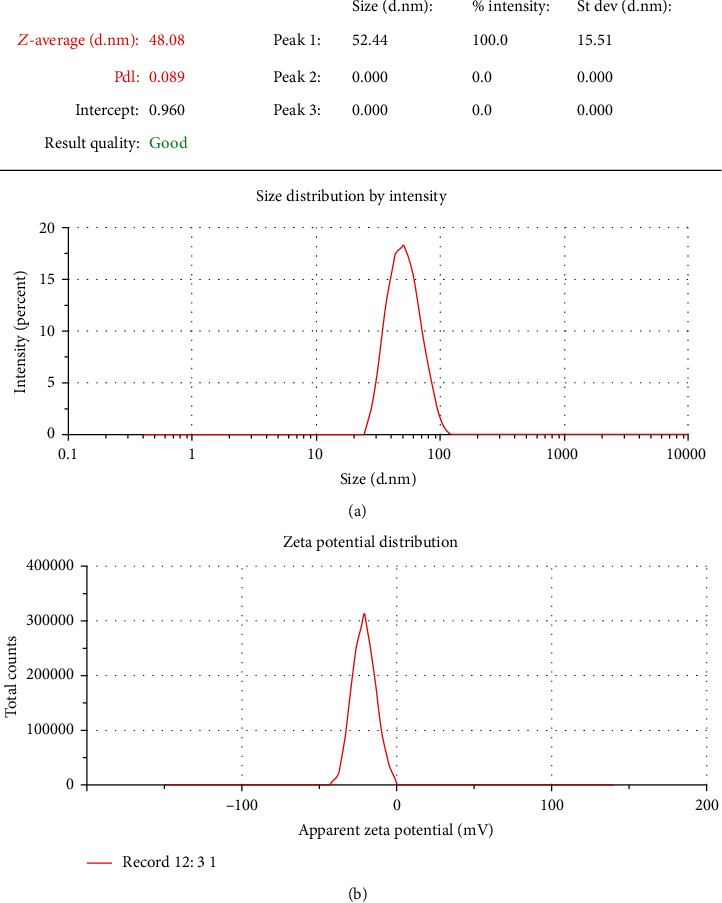
(a) DLS analysis of synthesis of Se NPs. (b) Zeta potential of synthesis of Se NPs.

According to the result of the DLS analysis, an average hydrodynamic diameter of synthesized Se NPs was 48 nm. The polydispersity index (PDI) in DLS analysis was 0.089 (Figure 2(a)) that confirmed the homogeneity and nondispersion of the nanoparticle size. Zeta potential of the nanoparticle was -21 mV which was detected by using a zeta sizer (Figure 2(b)).

**Figure 3:**
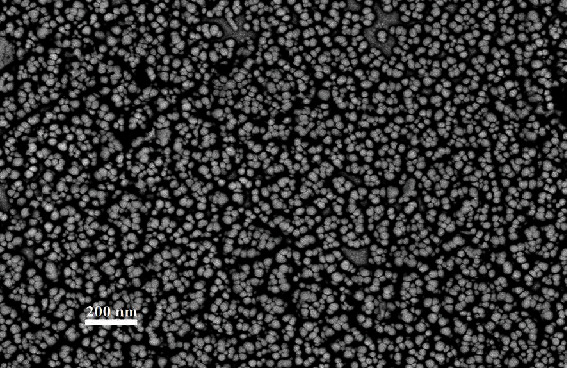
SEM image of synthesis of Se NPs.

The result of the SEM image indicated spherical morphology of nanoparticle shape with 34 nm in size. These results also confirmed the uniformity of the nanoparticles size and morphology (Figure 3).

**Figure 4:**
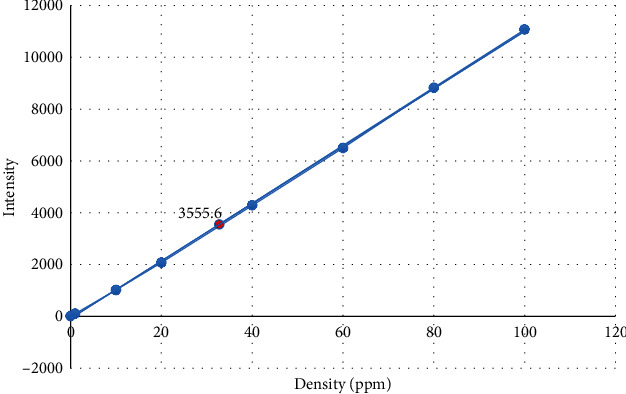
Calibration curve of concentration of standard selenium solutions.

In the ICP method, the calibration curve of the selenium standards was plotted and the concentration of the Se NPs was calculated as 0.654 *μ*g/ml (Figure 4).

### 3.2. Cytotoxic Effect of Se NPs

**Figure 5:**
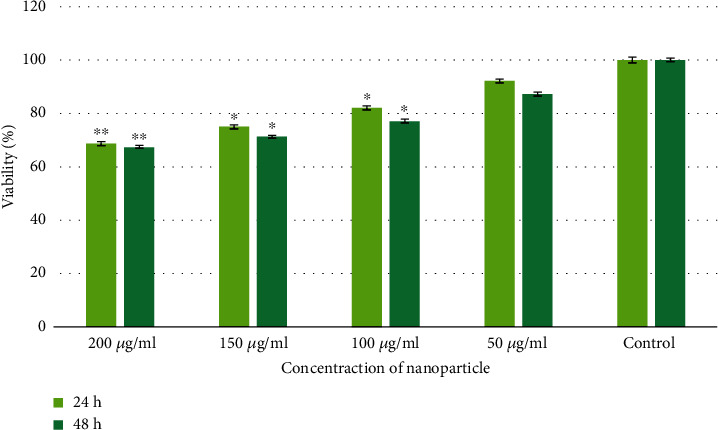
Viability of Caco-2 cells (^∗^*P* < 0.05, ^∗∗^*P* < 0.01).

The cytotoxic activity of Se nanoparticle on Caco-2 cells was analyzed by the MTT assay. As represented in Figure 5, in cells treated with 50-200 *μ*g/ml of Se NPs for 24 and 48 h, more than 50% of the cells were viable. Moreover, the viability of cells in 100 *μ*g/ml of Se NPs (concentration used in this study) after 24 and 48 h was 82.05% and 77.04%, respectively.

### 3.3. Antibody Responses

**Figure 6:**
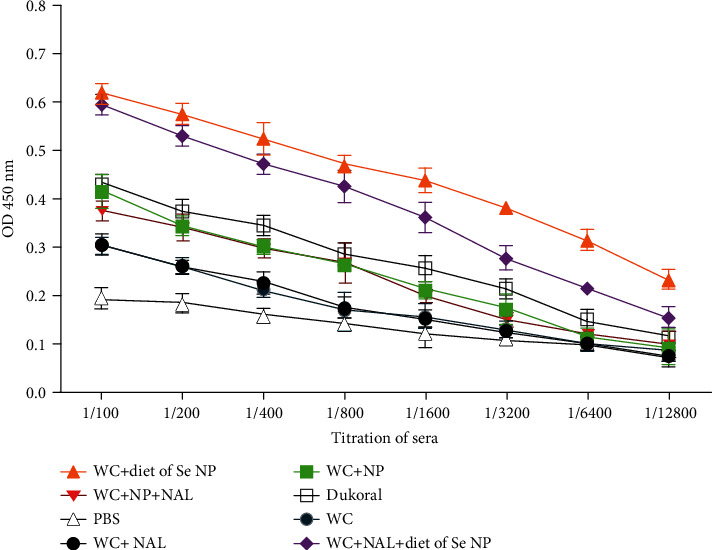
Titration of IgG antibody in sera in different immunized mouse groups on day 42.

ELISA was performed on serum and saliva samples 14, 28, and 42 days after immunization. The highest level of the antibody was observed on day 42. Following oral immunization, we found significant increase in IgG response in all immunized groups compared with the mice that only received PBS (*P* < 0.05). There has been a significant increase in IgG response in groups receiving selenium nanoparticle diet compared with whole-cell control group (*P* < 0.01). Moreover, in the diet groups, the IgG antibody level was significantly higher than that in the Dukoral vaccine group (*P* < 0.05). Moreover, a significant difference was observed in antibody responses of the group immunized with Se NPs—whole-cell group with the same group receiving an Se NP regimen (*P* < 0.05). In the mouse group that were immunized with whole cells in combination with naloxone, no substantial difference was observed in comparison with the whole-cell control group (*P* > 0.05) (Figure 6).

**Figure 7:**
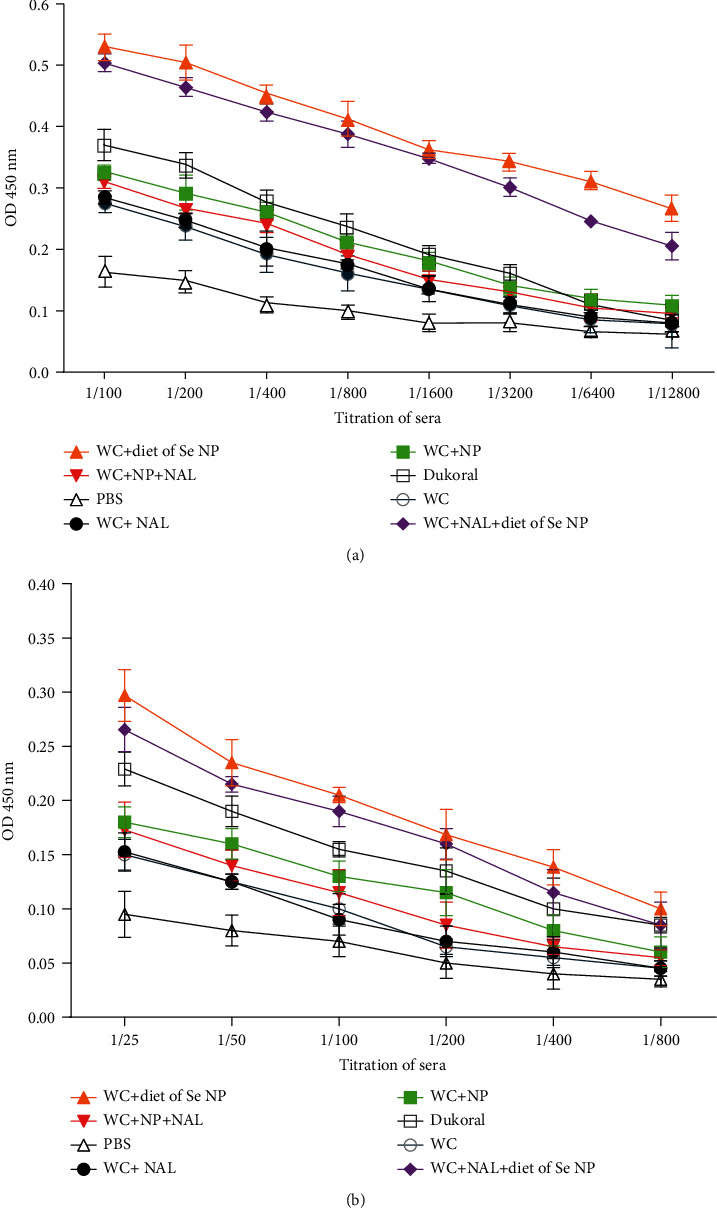
(a) IgA titer in serum of different mice on day 42. (b) IgA titer in saliva of different mice on day 42.

In the case of IgA antibody response, there was a significant antibody response in the serum sample of immune mice compared with the PBS control group (*P* < 0.05). In serum and saliva samples, much higher level of the IgA antibody was observed in groups with a selenium NP regimen (Figures 7(a) and 7(b)). A significant rise was detected in IgA level in the serum sample of mice with selenium diet groups in contrast with Dukoral vaccine and control groups (*P* < 0.05). No significant difference was detected between groups WC-Nal, WC-NP, WC-Nal-NP, and Dukoral compared with whole-cell alone group (*P* > 0.05). In saliva samples, a significant increase in IgA antibody response in the WC-diet of Se NP, WC-Nal-diet of Se NPs, and Dukoral vaccine group was detected in comparison with that in the whole-cell alone group (*P* < 0.05).

### 3.4. Measurement of Cytokine

**Figure 8:**
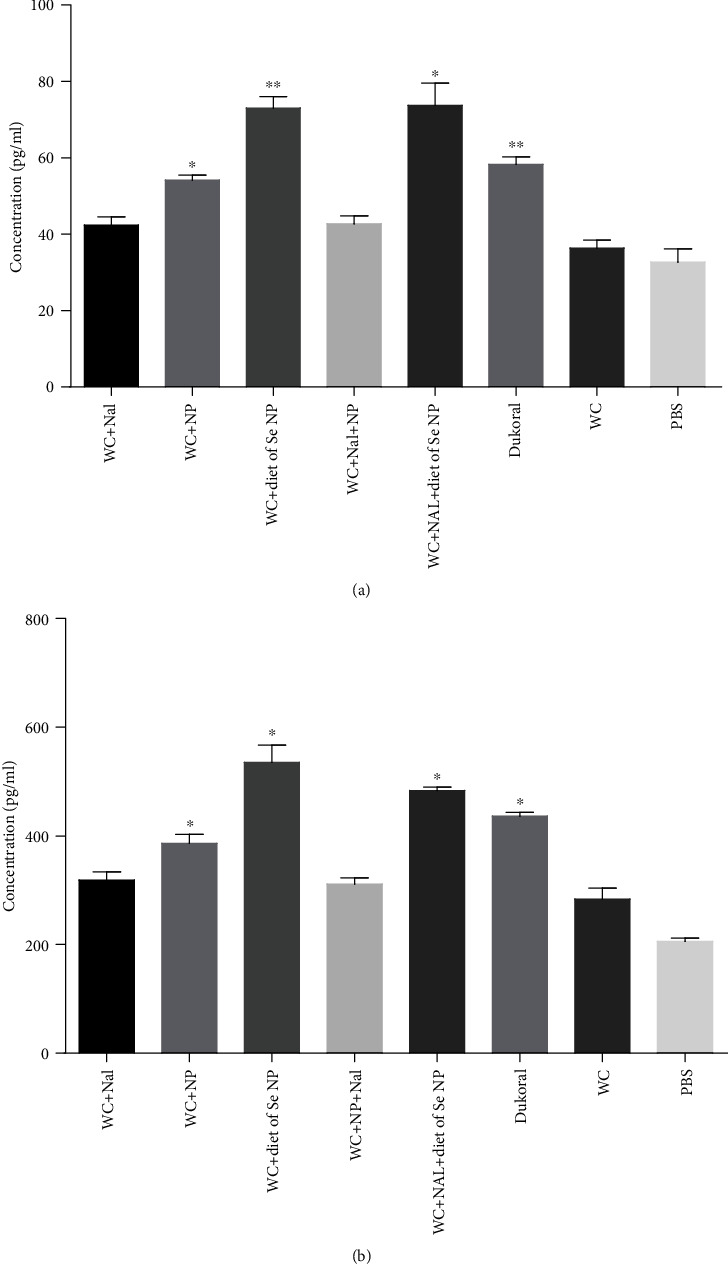
(a) Level of IL-4 in blood on day 42. Immune mouse groups have been compared with the whole-cell group. The data are presented as the mean ± SD (^∗^*P* < 0.05, ^∗∗^*P* < 0.01). (b) Level of IL-5 in blood on day 42. Immune mouse groups have been compared with the whole-cell group. The data are presented as the mean ± SD (^∗^*P* < 0.05).

Indirect ELISA was performed to investigate the level of IL-4 and IL-5 after immunization. The result indicated that IL-4 was increased in the serum sample of all immune mouse groups. In fact, two groups with selenium diet showed the highest level of IL-4, among which the WC-diet of Se NP group revealed significant increase compared with the Dukoral vaccine (*P* < 0.05) and whole-cell control groups (*P* < 0.01). Furthermore, in the immune mouse group, there was no significant disparity of IL-4 level in the whole-cell-Nal and whole-cell-Nal-NP groups in contrast with the whole-cell control group (*P* > 0.05) (Figure 8(a)). There was a significant increase in IL-5 level in mouse groups immunized with WC-Nal-NP diet of Se NPs compared with Dukoral vaccine (*P* < 0.05) and whole-cell control groups (*P* < 0.05) (Figure 8(b)).

### 3.5. Protection Assay

**Figure 9:**
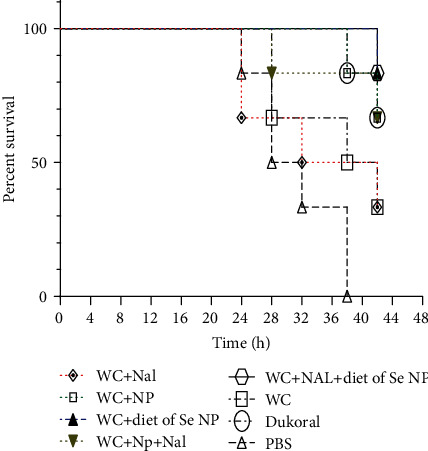
Percent survival of neonatal mice in challenge with whole-cell *V. cholerae*. Survival curves in neonate challenge were analyzed by the log-rank test (*P* < 0.001).

In neonatal mouse challenges, death was recorded every 4 h for 48 h, after gavage injection of compounds. No death was recorded within the first 24 hours. According to Figure 9, in the control group (PBS), mice started dying at the 24^th^ hour and all of them died during the 38^th^ hour. Overall, the death of the other groups started at 24, 28, and 38 h after injection, and all of them were more resistant than the control group.

Significant protection was observed against 1 LD50 V*. cholerae* in the group receiving diet of Se NPs (83.3% survival) compared with others. However, the WC-Se NP and WC-Se NP-Nal groups have the same survival rate as the Dukoral group (66.6% survival). The WC-Nal group like the whole-cell alone group showed a 33.3% survival rate (Figure 9) (*P* < 0.001).

## 4. Discussion

Two commercially available oral vaccines against *V. cholerae*, Dukoral and Shancol, are associated with killed whole-cell *Vibrio cholerae* [24]. These oral cholera vaccines have inclusive license, and the World Health Organization uses them globally in the endemic region, in parallel with improvements in sanitation and safe water that lead to cholera control and prevention [25, 26]. Dukoral is one of the oral cholera vaccines that contain killed *Vibrio cholerae* O1 classical and El Tor biotype whole cells together with the recombinant cholera toxin B subunit [27]. The vaccine is applicable in adults and children > 2 years of age with protective duration of about 50% over 3 years [28]. Shancol is a variant of whole-cell bivalent vaccine consisting of killed whole cells of *V. cholerae* serogroups O1 and O139 that is licensed for administration in adult and children older than 1 year [29, 30]. However, some studies on protective immunity of oral killed vaccine have suggested 79-86% protection for 6 months [31–33]. In fact, the protective immunity initiator by these vaccines is low-level and short-term [6]. Incorporation of adjuvants along with vaccine ingredients will increase the effect of the vaccine component and thus improve protective immunity. Dukoral vaccine has used an rCTB antigen as both immunogen and adjuvant, alongside the killed whole cells, and has improved the vaccine efficiency. Several antigens of *V. cholerae* have been studied as inducers of the immune system against bacteria, such as lipopolysaccharide, toxins, and outer membrane vesicles. Studies on cholera toxin reveal that these antigens create only a short-term protection if applied alone [5], and it is better to be used in combination with the killed whole-cell component. Other *V. cholerae* subunit antigens may elicit a good immune response as immunogen, but generally, their production requires lots of cost and a cold chain for storage and transportation. Despite extensive studies on different *V. cholerae* antigens as immunogens and their different efficacy, commercial vaccines against cholera still contain whole cells as the main component [24].

Immunoglobulin G (IgG) and secretory immunoglobulin A (sIgA) antibody responses to *V. cholerae* antigens play the most important role in protection. IgG antibodies specific for *V. cholerae* antigens are protective against cholera in the mouse model, and specific intestinal secretory IgA induced against *V. cholerae* creates the most protection [34]. Compared with parenteral killed whole-cell vaccines, oral vaccines provide long-term protection, and it is also worth noting that gastrointestinal immunity induces a secretory antibody that plays a key role in preventing bacterial attachment to the intestinal tract [34].

Research on modern vaccines with new ingredients could be useful, and it is proposed that it is better to use adjuvants in conjunction with vaccine compartment to increase the duration of protective immunity of oral vaccines. In a recent research on cholera nanovaccine, for example, gold nanoparticle has been used in conjugates with different *Vibrio cholerae* protective antigens and evaluated in two animal models (rabbit and mice), which showed high level of antitoxic antibodies and protective immunity compared with a common vaccine (control) [35]. Moreover, *Vibrio cholerae* lipopolysaccharide-loaded chitosan nanoparticles have been shown to induce a good immune response and high level of IgA, IgG, and IgM, along with a member of bacteria. These studies show that nanoparticles, in the role of an adjuvant or carrier, can be useful in inducing immune response in the cholera vaccine model [36].

In this study, two nonantigenic adjuvants, selenium nanoparticle and naloxone, were applied with killed whole cells, and their role in increasing the immune responses and protective efficacy of vaccine was investigated. The selenium nanoparticles synthesized and applied in this study were of uniform size with no agglomeration and little toxicity in vitro for Caco-2 cells. When selenium as an adjuvant was used in conjunction with WC, a significant increase was observed in mouse immune responses in comparison to the control group (WC), while in mice immunized with whole cell+daily diet of Se NPs, superior immune responses and protective efficacy were observed. In fact, administration of Se NP diet leads to significant increase in *V. cholerae*-specific IgG and IgA responses in serum and saliva and protective immunity in challenge experiment in comparison with control groups.

IL-4 and IL-5 were significantly increased in response to WC+daily diet of Se NPs with or without naloxone, and both of cytokines are supposed to be involved in activation, proliferation, and terminal differentiation of antigen-reactive IgA B cells to plasma cells and consequent antibody production. Moreover, IL-5 contributes to isotype switching of B cells to pIgA-producing plasma cells. This reveals that daily diet of Se NPs could efficiently induce immune cell effectors in both humoral and mucosal levels and increased immune responses due to daily diet of selenium NPs were even higher than those due to the commercial Dukoral vaccine; however, naloxone proved no effect on IL-4 and IL-5 increase and is proposed as null in the cytokine and antibody production process.

Previous studies have shown the inhibitory effects of selenium NPs on *V. cholerae* in vitro as well as their inhibitory effect on virulence gene expression of this bacterium [37]. The effect of selenium on the immune system is through molecular mechanisms; however, its precise pathways have not yet been fully established [38]. Actually, selenium plays an important role in immune system function, and its deficiency is associated with impaired innate and adaptive immune responses [38–40]. In a recent study by Mahdavi et al., the effect of selenium NPs as an adjuvant in the mouse model of hepatitis B vaccine was investigated. It was elucidated that selenium NPs were able to increase immune responses against the viruses [41], whereas we substantiate that Se NPs can also be effective as an adjuvant for the vaccine against extracellular bacterial pathogens. Recent studies have demonstrated that selenium nanoparticles in the role of an adjuvant have been able to induce good protective immune responses in the component with vaccine against bacteria and viruses [41–43]. In one study, selenium nanoparticles as an adjuvant in *Escherichia coli* antigens have been studied and showed that selenium nanoparticle can act as a good adjuvant in immunization [43]. In another research, the immunomodulatory effects of selenium NPs on breast cancer were investigated in a mouse model and it was shown that selenium nanoparticles can induce Th1 and cytokine responses [44, 45]. Therefore, it can be concluded that selenium nanoparticles as an immune booster can accompany vaccine components to increase the immune responses and to increase their efficacy in the control and prevention of cholera.

Surprisingly, we observed the slightest disparity in immune responses both in antibodies and in cytokines, in mice immunized with naloxone alone and together with Se NPs as an adjuvant for whole cells in comparison with the whole cells alone. Previous studies have shown that naloxone can shift the immune responses to a Th1 pattern and induce robust cellular immune responses against *herpes simplex virus type-1* and *Listeria monocytogenes* (intracellular pathogens) [13, 14]. However, our results indicated that naloxone as an adjuvant has not been able to adequately increase the immune system against *V. cholerae* and consequently resulted in lower protective immunity.

In conclusion, (i) Se NP can efficiently increase the protective efficacy of *V. cholerae* whole-cell compartment of cholera vaccine, (ii) Se NP as daily diet can more efficiently augment immune responses against *V. cholerae* whole cells, and (iii) naloxone revealed fair function in immunity against cholera.

## Figures and Tables

**Table 1:** Formulation of suspension for each group of mice.

Group	Suspension received
WC+Nal	250 *μ*l of PBS containing inactivated *Vibrio cholerae* (10^8^ CFU) and naloxone (0.15 mg)
WC+Se NPs	250 *μ*l of PBS containing inactivated *Vibrio cholerae* (10^8^ CFU) and selenium NPs (100 *μ*g)
WC+diet of Se NPs	250 *μ*l of PBS of inactivated *Vibrio cholerae* (10^8^ CFU), daily diet of Se NPs (100 *μ*g)
WC+Nal+Se NPs	250 *μ*l of PBS containing inactivated *Vibrio cholerae* (10^8^ CFU) and naloxone (0.15 mg) and selenium NPs (100 *μ*g)
WC+Nal+diet of Se NPs	250 *μ*l of PBS containing inactivated *Vibrio cholerae* (10^8^ CFU) and naloxone (0.15 mg), daily diet of Se NPs (100 *μ*g)
Dukoral vaccine	200 *μ*l of PBS containing Dukoral vaccine (10^8^ CFU)
Control	250 *μ*l of inactivated *Vibrio cholerae* (10^8^ CFU)
Control	250 *μ*l of PBS

## Data Availability

The datasets of the current study are available within the article or can be obtained from the corresponding author upon request.
